# Work-life balance mediating stress and quality of life in academics during COVID-19 in Malaysia

**DOI:** 10.4102/jphia.v15i1.562

**Published:** 2024-08-31

**Authors:** Lwin M. Aye, Jeremy Ern Hwei Tan, Shamala Ramasamy

**Affiliations:** 1 Department of Public Health and Community Medicine, School of Medicine, International Medical University, Kuala Lumpur, Malaysia; 2 School of Postgraduate Studies, International Medical University, Kuala Lumpur, Malaysia; 3 Department of Psychology, School of Psychology and Social Sciences, International Medical University, Kuala Lumpur, Malaysia

**Keywords:** perceived stress, work-life balance, quality of life, academicians, COVID-19 pandemic, Malaysia

## Abstract

**Background:**

Following the implementation of the Movement Control Order (MCO) during the coronavirus disease 2019 (COVID-19) pandemic, academicians from the universities in Malaysia needed to ensure that the quality-of-service delivery to the stakeholders is undisturbed by adopting new challenging norms. This compromises the work-life balance (WLB), causes more stress and potentially affects their quality of life (QoL).

**Aim:**

This study investigates how perceived stress (PS) impacts the QoL of Malaysian academicians during the COVID-19 pandemic, focusing on the mediating role of WLB.

**Setting:**

Academics working in Malaysia during COVID-19 pandemic.

**Methods:**

A cross-sectional study, using a voluntary response sampling method, was conducted among 417 academicians from universities in Malaysia in September 2021. A self-reported online questionnaire, measuring PS, WLB and QoL, was distributed.

**Results:**

The QoL scored a mean of 50 (standard deviation [s.d.] = 9.84), PS scored a mean of 24.26 (s.d. = 8.19) and WLB had a mean score of 51.12 (s.d. = 18.73). Work-life balance was a significant mediator of PS and QoL (β = –0.43, 95% confidence interval [CI] = –0.52 to –0.35, *p* = 0.0001). Perceived stress was a significant predictor of WLB (β = 1.62, *p* = 0.0001).

**Conclusion:**

Institutions should consider implementing flexible working arrangements, and providing workshops on crisis management, time management, and resilience. Stress coping methods are recommended for enhancing WLB among academicians.

**Contribution:**

This study contributes to the pool of evidence to support intervention strategies and policy recommendations aimed to enhance well-being.

## Background

The coronavirus disease 2019 (COVID-19) pandemic began in late 2019 and has profoundly impacted various aspects of life worldwide. One of the sectors significantly affected is academia, where educators and researchers have faced unprecedented challenges.^1^ The shift to online teaching, the pressure to maintain research productivity and the blurred boundaries between work and personal life have contributed to increased stress levels among academicians.^2,3^ In Malaysia, these challenges have been particularly pronounced because of the rapid transition to digital platforms and the societal expectations placed on educators.^4^

### Perceived stress

Perceived stress (PS) refers to the extent to which individuals feel overwhelmed or unable to cope with life’s demands.^5^ It is a subjective measure that reflects the imbalance between perceived demands and one’s ability to meet those demands. During the COVID-19 pandemic, academicians have experienced heightened levels of PS because of several factors. These include the rapid shift to online teaching without adequate preparation or training, the need to balance professional responsibilities with personal and family obligations and concerns about job security and health risks.^6,7^ According to a study conducted in Malaysia, 42.9% of academicians reported experiencing moderate to high levels of stress during the pandemic.^7,8^ The sudden and significant changes in the work environment have exacerbated feelings of stress and anxiety, potentially impacting both personal well-being and professional performance.^9^

### Quality of life

Quality of life (QoL) is a broad concept encompassing an individual’s overall well-being, including physical health, psychological state, level of independence, social relationships, personal beliefs and relationship to salient features of the environment.^10^ For academicians, the pandemic has disrupted many of these dimensions. The physical health of educators may be compromised because of prolonged hours of screen time and the lack of physical activity.^11^ Psychologically, the isolation from colleagues, students and the broader academic community has taken a toll, with findings of academicians reporting feelings of loneliness and isolation.^12^ Social relationships have also suffered, as interactions have become limited to virtual platforms, which can lack the richness of face-to-face communication. These disruptions can collectively contribute to stress diminishing the QoL.^13^

### Work-life balance

Work-life balance (WLB) is the equilibrium between professional work and personal life. It is a crucial aspect of overall well-being and job satisfaction.^14^ For academicians, maintaining a WLB has become increasingly challenging during the pandemic.^4^ The blending of home and work environments has led to longer working hours, with many educators reporting difficulties in setting boundaries between their professional and personal lives.^2,15^ A study revealed that employees found it challenging to separate work from personal life during the pandemic, leading to increased stress and decreased satisfaction with their WLB.^16,17^ The absence of a clear separation between work and home has resulted in an ‘always-on’ mentality, where academicians feel compelled to be available at all times for work-related tasks. This continuous connectivity can lead to burnout, reduced job satisfaction and a negative impact on QoL.^18^

### Relationship between stress, work-life balance, and quality of life

The relationship between PS and QoL is complex and influenced by various factors.^19^ One critical mediating factor is WLB. When WLB is disrupted, the stress experienced by a person can intensify, leading to further deterioration in their QoL.^20,21^ Conversely, effective management of WLB can mitigate the adverse effects of stress and promote better overall well-being. Research indicates that maintaining a balanced work-life is a significant predictor of overall QoL.^22^

### Impact of COVID-19 pandemic among the academicians

During the COVID-19 pandemic, Malaysia implemented its Movement Control Order (MCO) in March 2020 and the Ministry of Education have called a halt to conventional teaching and recommended a shift to online teaching (e-learning).^23^ With this sudden implementation, the private universities, which are profit-based, were also under substantial pressure to deliver the services paid for to the stakeholders undisturbed, but in a new way in a short amount of preparation time. The role of WLB has become even more significant. With the shift to remote work, academicians have had to develop new and unfamiliar strategies overnight and continue the service to the students amid concerns from the students and their guardians and carry out the duties of an academic while adapting to the new challenging situation within their own family resulting from the COVID-19 pandemic.^24,25^ Hence, with the stress because of the COVID-19 pandemic and other factors, this can affect the QoL of an individual. A person’s capability to perform at their best and their capacity to handle stressful situations will be impacted by their feeling of well-being, which results from pleasure or unhappiness with the aspects of life that are essential to them.

### Significance of the study

Understanding the interplay between PS, WLB and QoL among academicians during the COVID-19 pandemic has important implications for policy and practice. Institutions of higher education need to recognise the heightened stress levels and the potential for long-term impacts on the well-being of their staff. Developing policies that promote WLB, such as flexible working arrangements, mental health support and professional development opportunities, can help mitigate the negative effects of stress.

### Justifications and aims

Moreover, there is a need for research to explore the impact of the pandemic on the well-being of academicians who are working in private settings as the majority of the published studies focused on students during this challenging time.^13^ Such research can provide valuable insights into the effectiveness of different strategies and interventions aimed at improving WLB and overall QoL. By prioritising the well-being of academicians, private institutions can not only enhance individual outcomes but also foster a more resilient and productive academic community. Thus, this study aims to understand the mediating role of WLB and how it influences the intensity and direction of the association between PS and QoL among academicians in Malaysia.

## Methods

### Study setting and study population

The study was conducted among academicians from private universities in Malaysia. There are a total of 48 private universities. Any university that gave consent was selected to participate in the study. The study cohort comprised academicians who resided in Malaysia during the study period, were employed full time, and were proficient in the English language to respond to self-administered questionnaires.

### Study design, sampling and sample size

A cross-sectional study using a voluntary response sampling method was conducted by researchers from a Medical University in Kuala Lumpur, Malaysia, for 2 weeks in September 2021. Based on Malaysian statistics, it was estimated that 34 000 academicians were at the point of study. The Krejcie and Morgan^26^ chart was used, based on approximately 40 000 academicians, and a sample size of 380 participants was determined. The non-response rate of 10% was then considered, leading to a final sample size of 418 participants.

### Study instruments

#### Demographic factors

A structured self-administered questionnaire was used to collect the data from the participants. It consisted of four sections. Section A measured socio-demographic factors (5 items), section B measured PS (14 items), section C measured WLB (15 items) and section D measured QoL (16 items).

#### Perceived Stress Scale

Perceived stress was measured using the ‘Perceived Stress Scale’ (PSS), which was developed by Cohen et al. (1993).^5^ It comprises 14 items designed to measure the unpredictable, uncontrollable and overloaded individual’s perception of their life circumstances. The scale consists of a 5-point Likert scale with the scoring of 0 = Never, 1 = Almost Never, 2 = Sometimes, 3 = Fairly Often and 4 Very often. The scores ranged from 0 to 56 points, and a higher score indicates greater PS. Questions 4, 5, 6, 7, 9, 10 and 13 are reverse-scored. Perceived Stress Scale has an established Cronbach alpha of 0.78, indicating an acceptable reliability threshold.

#### Work-life balance

The WLB scale^27^ consists of 15 items with a 3-point semantic differential scale: ‘Not at all’ = 1, ‘Sometimes’ = 4 and ‘All the time’ = 7. The overall score ranges from 15 to 105. The scores are reversed for Questions 7, 12, 13, 14 and 15. A lower score indicates a higher level of WLB. The established Cronbach alpha is 0.89, which indicates a good measure of internal consistency.

#### Quality of life

Respondents’ QoL was measured by adopting the Quality-of-Life Enjoyment and Satisfaction Questionnaire (Q-LES-Q-SF), which was developed by Endicott et al.^28^ The scale measures an individual’s satisfaction and enjoyment in different areas of daily functioning. The questionnaire consists of 16 items with a 5-point Likert scale. (Very Poor = 1, Poor = 2, Fair = 3, Good = 4, Very Good = 5). The overall score ranges from 14 to 70 points. A higher score indicates greater life enjoyment and satisfaction. It has a Cronbach alpha of 0.900,^29^ indicating an excellent internal consistency measure.

Before the initiation of the study, a pilot study was carried out through an online survey among 30 academicians. Clarity, readability, feasibility and suitability of the questionnaire were checked. The feedback obtained from the participants was acknowledged and incorporated into the questionnaire for better sequence and clarity. The data from the pilot study was excluded from the data analysis.

### Data collection procedure

Based on the Malaysian Qualifications Register (MQR), 48 private universities were officially recognised, and a response from 10 universities was achieved. The participating universities are located in eight different states in Malaysia (Johor, Kedah, Kelantan, Malacca, Pahang, Perlis, Sabah and Terengganu).

Upon obtaining approval from universities, the self-administered questionnaires, which consisted of study information, consent seeking and eligibility self-screening on the first page, were distributed via mass emails using online platforms such as Google Forms. As this is a voluntary response survey, the data submitted by the participants were automatically recorded in google platform after they had confirmed their eligibility and given consent on the online form. Universities or participants that refused to participate were exempted.

### Data analysis plan

Statistical Package for the Social Sciences (SPSS) version 25.0 was used to analyse the data. Before the analysis, duplicate responses were removed by identifying the email addresses that made multiple responses. The characteristics of the participants are described by using descriptive statistics. The Baron and Kenny^30^ method of mediation analysis was adopted, and Hayes PROCESS Macro version 3.5 was used to analyse the mediating variable.

### Ethical considerations

Ethical clearance to conduct this study was obtained from the International Medical University (IMU), Kuala Lumpur, Malaysia (No. MSPHI/2020 [07]). This study does not contain any physical, psychological or social risks. Participation was strictly on a voluntary basis, and participants’ responses were kept anonymous according to the *University Data Protection Act*.

## Results

From the 10 private universities that agreed to participate across 8 states in Malaysia, emails were distributed to 2200, and a total of 427 participants were obtained. Out of these, 10 participants were excluded because of questionable errors and incomplete responses to the questionnaire.

### Characteristics of the respondents

The respondents were with a mean age of 44 years and a standard deviation of 11.68. The sample consists of predominantly female (*n* = 270, 64.7%) and Malaysian nationals (*n* = 347, 83.2%), with the Chinese ethnicity comprising the majority (*n* = 175, 42%). Most participants were married (*n* = 308, 73.8%).

The mean scores for the outcome, independent factor and mediator are described in Table 1. The QoL scored a mean of 50 (standard deviation [s.d.] = 9.84, range = 14–70), PS scored a mean of 24.26 (s.d. = 8.19, range = 2–48) and WLB had a mean score of 51.12 (s.d. = 18.73, range = 15–105). Assumptions of normality were not violated.

**TABLE 1:** Mean scores of quality of life, perceived stress and work-life balance.

Variables (continuous variable)	Total survey (*N* = 417)		
	Mean ± s.d.	Minimum	Maximum
Quality of life	50.00 ± 9.48	14.00	70.00
Perceived stress	24.26 ± 8.19	2.00	48.00
Work-life balance	51.12 ± 18.73	15.00	105.00

s.d., standard deviation.

### Mediating effects of work-life balance between perceived stress and quality of life

As per the model summary presented in Table 2 and Table 3, depicting the effects of PS on WLB, it is evident that PS is a significant predictor of WLB (beta coefficient = 1.62, *p*-value = 0.0001), exhibiting a positive relationship.

**TABLE 2:** Results for model summary: Direct and Indirect effects of PS on WLB.

Variable	Beta coefficient	s.e.	*t*-value	*p*
**Model summary of effects of PS on WLB**				
Total PS score	1.62	0.08	20.43	0.0001*
**Model summary of an indirect effect of PS on WLB**				
Total PS score	−0.30	0.05	−5.57	0.0001*
Total WLB	−0.27	0.02	−11.27	0.0001*

QoL, quality of life; PS, perceived stress; WLB, work-life balance; s.e., standard error.

* , *p*-value less than 0.05 is considered statistically significant.

**TABLE 3:** Results for the mediation effects of work-life balance between perceived stress and QoL.

Variable	Effect	s.e.	*t*-value	Lower 95% CI	Upper 95% CI
**Total effects of PS on WLB**					
Total PS score	−0.74	0.044	−16.80	−0.82	−0.65
**Indirect effects of PS on QoL through WLB**					
Total WLB score	−0.43	0.045	-	−0.52	−0.35

QoL, quality of life; PS, perceived stress; WLB, work-life balance; s.e., standard error; CI, confidence interval.

The same table provides a model summary of the indirect effect of PS on QoL. Perceived stress is identified as a negative predictor of QoL with a beta coefficient of –0.30 and a significant *p*-value of 0.0001. The WLB scale also serves as a significant negative predictor of QoL, presenting a beta coefficient of –0.27 with a *p*-value of 0.0001.

Table 2 and Table 3 further reveals the total effects of PS on WLB, showcasing a negative effect value of –0.74, standard error (s.e.) = 0.044. The 95% confidence interval (CI), spanning from –0.82 to –0.65, substantiates the statistical significance of the effect.

Examining the indirect effects of PS on QoL with WLB as a mediating variable, WLB demonstrates a negative effect value of –0.43, s.e. = 0.045. The 95% CI, ranging from –0.52 to –0.35, underscores the statistical significance of the effect. Consequently, the results suggest that higher PS correlates with lower WLB scores, implying better WLB (where a higher WLB score corresponds to poorer WLB and vice versa).

Upon controlling for the mediator, the direct effect of PS on QoL diminishes, indicating a mediation effect. However, the direct effect remains significant, signifying partial mediation.

The proportion of the total effect of PS on QoL that operates indirectly is calculated as 58.11% (indirect effect divided by total effect), indicating that 58.11% is mediated through WLB. The remaining 41.89% of the relationship operates directly, suggesting that PS directly impacts QoL. This analysis confirms partial mediation, highlighting the negative effects of PS on QoL, wherein WLB mediates these effects and exacerbates the negative impact on QoL. The higher the PS, the lower the WLB and QoL among academicians.

## Discussion

This study investigated the relationship between PS and QoL among Malaysian academicians during the COVID-19 pandemic, examining the mediating role of WLB. The study involved 417 participants from 10 private universities across 8 states in Malaysia. The majority of respondents were female (64.7%), Malaysian nationals (83.2%), primarily of Chinese ethnicity (42%) and married (73.8%), with an average age of 44 years.

The findings revealed a significant negative relationship between PS and QoL, indicating that higher levels of stress were associated with lower QoL. This finding aligns with existing literature highlighting the detrimental effects of stress on overall well-being.^31,32^ The study also found a significant positive relationship between PS and WLB, suggesting that higher stress levels were linked to poorer WLB. This finding supports previous research indicating that stress can negatively impact individuals’ ability to manage work and personal life demands effectively.^33,34,35^

Importantly, the study’s mediation analysis demonstrated that WLB partially mediated the relationship between PS and QoL. Specifically, 58.11% of the total effect of PS on QoL was mediated through WLB. This finding suggests that higher PS leads to poorer WLB, which, in turn, contributes to lower QoL. This finding highlights the crucial role of WLB in mitigating the negative impact of stress on well-being, particularly among academics who may face unique challenges in maintaining a healthy WLB.

Several factors unique to the Malaysian context and the COVID-19 pandemic might have contributed to the observed findings. The abrupt shift to online teaching, increased workload and blurred boundaries between work and personal life during the pandemic likely exacerbated stress levels and challenged WLB among academicians.^36,37^ Additionally, cultural factors in Malaysia, such as societal expectations and family obligations, might influence individuals’ experiences of stress and their ability to achieve WLB.^38,39,40,41^

The study’s findings have important implications for academicians and higher education institutions in Malaysia. Universities should prioritise initiatives that promote WLB and mitigate stress among their staff. Such initiatives could include:

- *Flexible work arrangements*: Offering flexible work schedules, remote work options and generous leave policies can help employees better manage their work and personal responsibilities.
- *Stress management programmes*: Providing access to counselling services, stress reduction workshops and mindfulness training can equip employees with coping mechanisms to manage stress effectively.
- *Work-life balance training*: Workshops and resources that educate employees on strategies for setting boundaries, prioritising tasks and improving time management skills can empower them to achieve a healthier WLB.

Further research could explore the effectiveness of these interventions in improving well-being among Malaysian academicians. Additionally, future studies could investigate the role of other potential mediators, such as coping strategies and social support, in the relationship between PS, WLB and QoL.

### Limitations

Even though this study is the first study conducted among academicians in Malaysia to observe the mediating role of WLB between PS and QoL, it has some limitations. Firstly, as this is a cross-sectional study, causality cannot be established. Secondly, as the non-probability sampling method was deployed because of challenges in getting the exact sampling frame, the generalisability of the results to the larger academic population is questionable. Thirdly, the results from the self-administered questionnaire may impose self-selection bias or recall bias, potentially diluting the validity of our findings.

## Conclusion

This study focused on the relationship between PS and QoL, mediated by WLB among academicians during the COVID-19 pandemic. Findings suggested that there was a significant negative relationship between PS and QoL. Work-life balance acted as a significant mediator between PS and QoL among academicians during the unprecedented pandemic. The organisations need to recognise and address job aspects that might affect employees’ capacity to effectively balance their work and personal lives. It is vital to acknowledge the relationship between PS and the QoL of academicians as it has implications for their mental well-being, thus, ensuring that the recommendations are considered to improve WLB and overall mental well-being.
